# Vision-Based Quality Grading of Beef Steaks Using Marbling Distribution Analysis and Lean Meat Color Classification

**DOI:** 10.3390/s26123812

**Published:** 2026-06-15

**Authors:** Hong-Dar Lin, Rong-Lun Chung, Chou-Hsien Lin

**Affiliations:** 1 Department of Industrial Engineering and Management, Chaoyang University of Technology, Taichung 413310, Taiwan; s10515608@cyut.edu.tw; 2 Department of Civil, Architectural and Environmental Engineering, The University of Texas at Austin, Austin, TX 78712-0273, USA; chslin@utexas.edu

**Keywords:** vision-based inspection, beef steak quality grading, fat marbling distribution, lean-meat color analysis, curvelet transform, chi-square goodness-of-fit test, support vector machine

## Abstract

This study proposes a vision-based framework for automated inspection and quality grading of beef steaks by integrating fat marbling distribution analysis and lean-meat color evaluation. In frozen beef products, surface frost often generates specular reflections that resemble both fat and lean regions, thereby reducing segmentation accuracy. To address this challenge, a sequential and interpretable analytical framework is developed. First, homomorphic filtering is applied to suppress frost-induced illumination artifacts, followed by curvelet transform combined with square-ring filtering to separate fat and lean regions based on their multi-scale and directional characteristics. For marbling analysis, the convex hull, skeleton, and principal axis of the steak are extracted, and a chi-square goodness-of-fit test is performed within eight predefined regions to quantitatively evaluate marbling distribution uniformity and identify localized fat accumulation. For lean-meat evaluation, RGB color features are extracted and classified using a Support Vector Machine (SVM) to determine redness levels. The resulting marbling and color information are subsequently integrated through a weighted grading strategy to estimate the final quality grade. Experimental results demonstrate a fat detection rate of 92.68%, a false-positive rate of 4.97%, and a correct classification rate of 94.09% for fat segmentation, while the SVM-based lean-meat color classifier achieves an accuracy of 96.67%. Furthermore, the proposed grading framework attains an overall grading accuracy of 90.38%, showing strong agreement with human evaluation.

## 1. Introduction

Beef quality assessment is critical in the meat industry, as it directly influences consumer preference and market value. Among various indicators, fat marbling distribution and lean meat color are key determinants of steak quality [1]. Traditionally, these attributes are evaluated by trained inspectors; however, such manual assessment is subjective, time-consuming, and prone to inconsistency. With advances in computer vision and machine learning, automated inspection systems have emerged as effective tools for objective and consistent quality evaluation by quantitatively analyzing visual features [2].

Despite this progress, several challenges remain, particularly for frozen beef. Frost formation on the steak surface introduces strong reflections and visual artifacts that resemble both fat and lean tissue, complicating accurate segmentation [3,4]. Conventional spatial-domain methods often fail to distinguish these artifacts, leading to unreliable marbling analysis [5]. Moreover, uneven fat distribution and localized accumulation—important quality indicators—are rarely quantified using rigorous statistical methods. Variations in illumination and surface texture further complicate lean meat color classification. These issues highlight the need for a robust and interpretable analysis framework.

To address these challenges, this study proposes a vision-based system for automated beef quality grading. Homomorphic filtering is applied to suppress frost and illumination effects, followed by curvelet transform with square-ring filtering to segment fat and lean regions. Marbling distribution is quantitatively evaluated by dividing the steak along its principal axis and applying a chi-square goodness-of-fit test to detect fat accumulation. Lean meat color is assessed using RGB features and classified via a Support Vector Machine (SVM). The final quality grade is obtained by integrating marbling uniformity and color evaluation.

The main contributions are (1) a unified framework combining marbling distribution and color analysis; (2) a frequency-domain preprocessing method for frost suppression; (3) a curvelet-based segmentation approach for accurate fat extraction; (4) a statistical method for quantifying marbling uniformity; and (5) an SVM-based lean color classification integrated into overall grading.

The remainder of this paper is organized as follows. Section 2 reviews the related work on vision-based meat quality assessment. Section 3 presents the materials and proposed methodology in detail. Section 4 describes the experimental setup and results, including comparative analyses with conventional and deep learning methods, followed by a discussion of the method’s effectiveness, robustness, and limitations. Finally, Section 5 concludes the study and outlines potential directions for future research.

## 2. Related Work

In recent years, significant research efforts have been devoted to developing automated systems for beef quality evaluation using computer vision and machine learning technologies. These systems aim to improve the objectivity, efficiency, and consistency of traditional manual grading processes. Various approaches have been proposed to analyze key visual attributes of beef, including marbling distribution, meat color, and texture characteristics. Existing research can generally be categorized into several major directions, including traditional computer vision methods for marbling segmentation, machine learning-based color classification techniques, advanced spectral imaging approaches, and, more recently, deep learning-based inspection systems. This section reviews representative studies in these areas and discusses their advantages and limitations in the context of automated beef steak quality grading.

### 2.1. Traditional Beef Quality Evaluation and Early Automation Attempts

Beef quality grading plays an essential role in the meat industry because it directly influences product value, consumer purchasing decisions, and supply chain standardization. Traditionally, beef grading has relied on manual visual inspection conducted by trained graders who evaluate several attributes, including marbling distribution, lean meat color, fat color, texture, and firmness according to established grading systems such as USDA or AUS-MEAT standards. Although this approach has been widely adopted, manual inspection is inherently subjective and may produce inconsistent grading results due to differences in grader experience, fatigue, and environmental conditions. Consequently, researchers have long sought to develop automated inspection systems capable of providing objective and repeatable evaluation of beef quality.

Early efforts toward automated beef grading primarily focused on computer-assisted image analysis techniques. Yoshikawa et al. proposed one of the earliest automated beef marbling grading systems using discriminant threshold selection and run-length processing to quantify marbling patterns from digital images [6]. Their work demonstrated that image processing techniques could effectively segment fat and lean regions and extract quantitative descriptors of marbling structure [7]. However, subsequent studies reported that image-based grading systems could be affected by variations in lighting conditions, surface reflections, and imaging noise, which may reduce segmentation accuracy and grading reliability [8]. These challenges motivated further research into more robust computer vision methods for beef quality inspection.

### 2.2. Computer Vision-Based Marbling Segmentation and Grading

With the advancement of digital imaging technology, computer vision has become one of the most widely used approaches for automated beef quality evaluation. Computer vision systems enable rapid, non-destructive, and objective analysis of meat characteristics by extracting visual features such as color distribution, texture patterns, and marbling structures from digital images. Several studies have demonstrated that machine vision can effectively quantify marbling attributes and predict beef quality grades [9,10].

Many existing computer vision approaches focus on marbling segmentation, which is considered one of the most important indicators for beef quality grading. Traditional methods often employ thresholding techniques, morphological operations, and texture feature analysis to separate fat tissues from lean muscle areas in RGB images [11]. For example, Barbon et al. developed a flexible computer vision system capable of classifying marbling grades under varying imaging conditions by combining image preprocessing, feature extraction, and machine learning models [12]. Their study demonstrated that machine vision could provide reliable marbling classification results when appropriate feature descriptors and classification algorithms are employed.

In addition to stationary imaging systems, portable computer vision devices have also been developed to facilitate practical deployment in meat processing facilities. Cardenas et al. proposed a portable electronic marbling evaluation system based on RGB image analysis and machine learning algorithms, which achieved high agreement with expert grading results according to established marbling standards [13]. These systems highlight the potential of computer vision technologies for real-time industrial applications. Nevertheless, most existing methods primarily estimate overall marbling quantity or marbling score, while relatively few studies investigate the spatial distribution or uniformity of marbling patterns, which are also important indicators of meat quality.

### 2.3. Meat Color Evaluation Using Image Analysis and Machine Learning

In addition to marbling characteristics, meat color is another critical quality attribute that significantly influences consumer perception and purchasing decisions. Fresh beef is typically expected to exhibit a bright cherry-red color, which indicates desirable quality and freshness. Consequently, objective color evaluation has become an important research topic in automated meat inspection systems.

Computer vision methods commonly extract color features from different color spaces, such as RGB, HSV, or CIE Lab, to quantify meat color characteristics and perform classification tasks. Chen et al. investigated the use of machine vision combined with support vector machines (SVM) to classify beef fat color grades and demonstrated that machine learning models can achieve high classification accuracy when appropriate color features are used [14]. Similarly, Sánchez et al. employed multivariate analysis and machine learning techniques to analyze beef color characteristics and predict quality attributes based on extracted image features [15].

Despite these advances, many existing studies focus on fat color evaluation or freshness-related discoloration, rather than directly assessing lean meat color grades as required for integrated beef quality grading. Moreover, color analysis is often performed independently of marbling analysis, resulting in separate evaluation systems that do not fully capture the combined visual attributes influencing overall beef quality.

### 2.4. Advanced Optical Imaging and Spectral Analysis

Beyond conventional RGB imaging, advanced optical sensing technologies have also been explored to improve the accuracy of beef quality evaluation. Among these techniques, hyperspectral imaging has attracted considerable attention because it combines spatial imaging with spectral analysis, allowing detailed characterization of the chemical and structural properties of meat tissues. Hyperspectral imaging systems capture reflectance spectra across a wide range of wavelengths, enabling more precise discrimination between fat and lean tissues compared with conventional color cameras.

ElMasry et al. reviewed the application of hyperspectral imaging for meat quality evaluation and highlighted its potential for detecting compositional differences and predicting quality attributes [16]. Velásquez et al. further demonstrated that hyperspectral imaging combined with decision-tree classifiers could effectively classify beef marbling grades [17]. More recently, structured illumination reflectance imaging (SIRI) has also been investigated to enhance texture and structural information in meat images, thereby improving marbling detection performance [18].

Although these advanced optical imaging techniques provide improved analytical capabilities, they often require specialized equipment, complex calibration procedures, and higher implementation costs. As a result, their adoption in routine industrial environments remains limited compared with conventional RGB-based machine vision systems.

### 2.5. Deep Learning-Based Beef Grading Systems

In recent years, deep learning has emerged as a powerful tool for automated visual inspection and has been increasingly applied to meat quality evaluation. Convolutional neural networks (CNNs) and object detection architectures have demonstrated strong performance in image recognition tasks by automatically learning hierarchical feature representations from raw images. Several recent studies have applied deep learning techniques to beef marbling detection and grading.

For example, Liu et al. proposed a beef marbling grading algorithm based on an improved YOLOv8x model incorporating attention mechanisms to enhance feature extraction and classification performance [19]. Deep learning models can significantly improve prediction accuracy when large annotated datasets are available and imaging conditions are well controlled [20]. However, these approaches typically require extensive training data and computational resources, and their performance may degrade when imaging artifacts or environmental variations occur.

### 2.6. Research Gap and Motivation

Despite significant advances in automated beef quality inspection, several critical challenges remain. Firstly, many existing computer vision systems are developed for fresh beef images captured under controlled laboratory conditions; consequently, their performance often degrades in real-world scenarios where surface artifacts such as frost formation and specular reflections are present. Secondly, prior studies predominantly focus on estimating overall marbling quantity or predicting marbling scores, while relatively few investigate the spatial distribution and uniformity of marbling patterns or explicitly detect localized fat accumulation defects—both of which are important indicators of meat quality. Thirdly, color-based analyses are typically conducted independently and often emphasize fat color or freshness, rather than integrating lean meat color grading with marbling characteristics.

These limitations highlight a clear research gap: the lack of a unified and robust inspection framework capable of handling imaging artifacts while jointly analyzing structural and visual quality attributes. In particular, there is a need for methods that can (i) suppress frost-induced interference, (ii) accurately segment fat and lean regions, (iii) quantitatively evaluate marbling distribution using statistical measures, and (iv) simultaneously classify lean meat color for comprehensive quality grading.

To address the aforementioned challenges, this study proposes a vision-based beef quality grading framework that integrates frequency-domain image preprocessing, curvelet-based marbling segmentation, statistical analysis of fat distribution, and machine-learning-based lean-meat color classification. Unlike conventional approaches that focus primarily on marbling quantity estimation or end-to-end classification, the proposed framework is designed as a sequential and interpretable analytical pipeline in which each component addresses a specific challenge in frozen beef inspection. Specifically, homomorphic filtering is employed to suppress frost-induced illumination artifacts and specular reflections; curvelet transform combined with square-ring filtering is used to accurately extract marbling structures; chi-square goodness-of-fit analysis quantitatively evaluates marbling distribution uniformity and identifies localized fat accumulation; and SVM-based classification objectively assesses lean-meat color characteristics. These complementary analyses are subsequently integrated to provide a comprehensive quality-grading framework that combines structural and color information. Therefore, the novelty of this work lies not only in the individual techniques employed but also in their systematic integration to simultaneously achieve frost suppression, marbling segmentation, marbling uniformity evaluation, and lean-meat color grading within a unified and interpretable framework. This integrated approach enables more reliable, explainable, and practically applicable beef quality assessment under realistic imaging conditions. The detailed methodology is presented in the following section.

## 3. Materials and Methods

The objective of the proposed steak quality grading system is to accurately evaluate beef steak quality by jointly analyzing marbling distribution and lean-meat color characteristics from digital images. The overall processing pipeline consists of several stages, including image acquisition, image preprocessing, marbling segmentation, marbling distribution analysis, lean meat color classification, and final quality grading. First, frozen beef steak images are captured using a digital imaging setup and resized to a uniform resolution for further processing. Image preprocessing is then applied to suppress frost artifacts and reduce illumination variations on the steak surface. Subsequently, the curvelet transform combined with square-ring filtering is employed to segment fat and lean meat regions from the steak images. Based on the segmented fat regions, the spatial distribution of marbling is analyzed using geometric structure extraction and statistical testing to identify potential fat accumulation defects. In parallel, RGB color features extracted from the lean meat regions are classified using a Support Vector Machine (SVM) to determine the lean meat color grade. Finally, the results from marbling analysis and color classification are integrated to estimate the overall quality grade of the beef steak. The detailed procedures of each component are described in the following subsections.

### 3.1. Image Acquisition and Beef Steak Samples

To ensure that the experimental conditions closely resemble real industrial inspection scenarios, the frozen beef short-rib steak samples used in this study were obtained from a commercial beef supplier located in central Taiwan. Throughout the remainder of this paper, these samples are collectively referred to as beef steaks for consistency of presentation. The samples consisted of commercially available frozen beef steaks and were used as the inspection objects in this study. Image acquisition was conducted in a laboratory environment after the samples were transported from a freezer maintained at −10 °C. During transport, the samples were placed in insulated cooling bags with ice packs to prevent damage or degradation in quality. Since the laboratory temperature was maintained at approximately 20–22 °C, frost formed on the surfaces of the beef samples due to temperature differences during handling and imaging.

To simulate inspection conditions commonly found in beef processing lines, each steak sample was placed on a flat platform and tilted at an angle of 45° during image acquisition. A color CCD industrial camera was used for image capture. Illumination was provided by a direct ring light source, which produced uniform lighting distribution across the sample surface. The CCD camera was positioned at the center of the ring light to ensure consistent illumination and minimize shadow effects. The light intensity was carefully adjusted to enhance the contrast between fat marbling and lean meat regions. In addition, black velvet cloth was placed beneath the samples to reduce specular reflections from the background.

**Figure 1:**
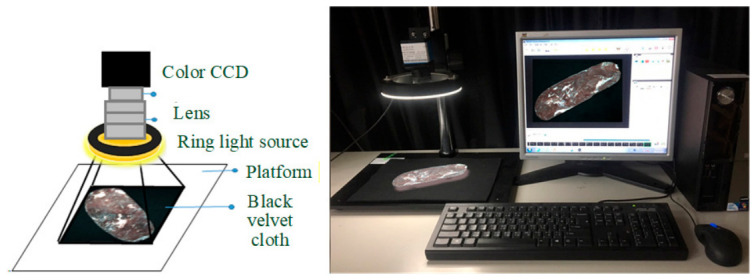
Experimental hardware configuration for image acquisition in this study.

**Figure 2:**
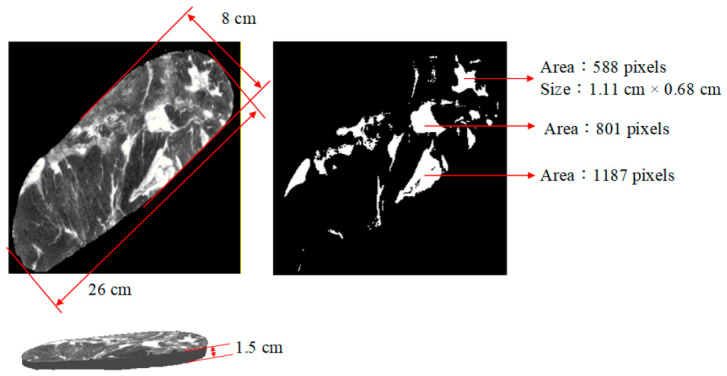
Dimensional diagram of the sample to be tested.

The imaging area for each capture covered approximately 20 cm × 14 cm, and the illumination was evenly projected onto the sample surface to ensure clear image acquisition. The experimental hardware configuration used for image acquisition is illustrated in Figure 1. In this study, the detection threshold for fat accumulation defects was defined as fat regions larger than approximately 1 cm in length and 0.5 cm in width (equivalent to about 500 pixels), as shown in the top-view illustration in Figure 2. In addition, the thickness of the steak samples when placed flat during imaging was approximately 1.5 cm, as illustrated in the front-view diagram in Figure 2.

A total of 1050 beef steak images were collected for model development, parameter optimization, and performance evaluation. To avoid bias caused by class imbalance, the dataset was designed with an equal number of samples for each quality grade. The dataset was partitioned into independent training, validation, and testing subsets following a 60%/20%/20% split. The training set was used for model development, the validation set for parameter optimization, and the testing set for final performance evaluation. The detailed class distribution is summarized in Table 1.

### 3.2. Noise Removal Using Homomorphic Filtering

During the experiments, the beef samples were captured under frozen conditions, which caused frost to form on the steak surface when exposed to ambient air. Under illumination, these frosted regions produce strong specular reflection artifacts, which interfere with subsequent image processing and make it difficult to accurately distinguish fat from lean meat regions. Therefore, an effective preprocessing step is required to reduce illumination-related noise and improve the visibility of intrinsic texture information.

In this study, homomorphic filtering [21] is employed to suppress frost-induced reflection noise. Based on the illumination–reflectance model, an image *f*(*x*,*y*) can be expressed as

(1) f(x,y)=i(x,y) r(x,y),

where *i*(*x*,*y*) represents the illumination component and *r*(*x*,*y*) represents the reflectance component. Since the illumination component mainly corresponds to low-frequency information and the reflectance component mainly contains high-frequency details, the logarithm of the image is first taken to convert the multiplicative relationship into an additive form:

(2) $$\ln f(x,y)=\ln i\left(x,y\right)+\ln r\left(x,y\right).$$

The transformed image is then mapped into the frequency domain using the Fourier transform:

(3) Z(u,v)=F[lnf(x,y)].

A homomorphic filter $H\left(u,v\right)$ is subsequently applied to attenuate low-frequency illumination variations and enhance high-frequency reflectance details:

(4) $$S\left(u,v\right)=H\left(u,v\right)\;Z\left(u,v\right).$$

In this study, the homomorphic filter is defined as

(5) $$H\left(u,v\right)=\left(h-l\right)\left\{1-exp\left[-c\;\frac{D^{2}\left(u,v\right)}{D_{0}^{2}}\right]\right\}+l,$$

where h and l denote the high-frequency gain and low-frequency gain, respectively, c is a constant controlling the sharpness of the filter transition, $D\left(u,v\right)$ is the distance from the frequency point $\left(u,v\right)$ to the center of the frequency domain, and $D_{0}$ is the cutoff frequency. After filtering, the processed image is transformed back into the spatial domain by the inverse Fourier transform, followed by the exponential operation:

(6) $$g\left(x,y\right)=exp\left\{F^{-1}\left[S(u,v)\right]\right\}.$$

In this study, the high-frequency gain (h) is set to 1.5, and the low-frequency gain (l) is set to 0.5. These settings are selected to enhance fine-texture details while suppressing uneven illumination and frost-related reflection effects.

**Figure 3:**
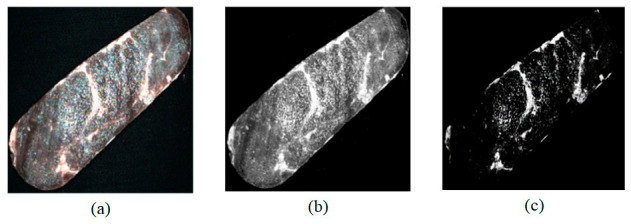
Effect of homomorphic filtering on frost removal from the beef steak surface: (**a**) original image; (**b**) grayscale image; and (**c**) defrosted image after homomorphic filtering.

As shown in Figure 3a,b, the original images exhibit pronounced reflection artifacts due to surface frost. In contrast, Figure 3c shows that, after homomorphic filtering, the reflection noise is significantly reduced and the fat-related structural features become more distinguishable. Therefore, homomorphic filtering is an effective preprocessing step that improves image quality and enhances the reliability of subsequent fat segmentation and quality analysis.

### 3.3. Enhancement of Fat Texture Using Curvelet Transform with Square-Ring Filtering

The curvelet transform, introduced by Emmanuel Candès and David Donoho in 1999 [22], is a multiresolution geometric analysis tool particularly effective at representing curvilinear structures [23]. Unlike traditional wavelet transforms, which have limited directional selectivity, the curvelet transform provides high directional sensitivity and anisotropic scaling, making it well-suited to capture edge and texture information in complex structures such as fat marbling patterns in beef. Moreover, the curvelet transform enables sparse representation of curved features in the transform domain, avoiding the mixed-frequency artifacts commonly observed in Fourier or wavelet representations. This property makes it highly suitable for enhancing fat texture patterns before segmentation.

#### 3.3.1. Frequency-Domain Decomposition via Curvelet Transform

In practical implementation, the curvelet transform is performed using the wrapping-based approach, where the image is mapped into localized affine regions in the frequency domain. The curvelet coefficients are denoted as *c^D^*(*j*, *l*, *k*), where *j*, l, and *k* represent the scale, orientation, and spatial position, respectively. The decomposition process is summarized as follows:

Step 1: Input an image *f*(*x*,*y*) of size N × N, and compute its 2D Fourier transform:

(7) F[u,v]=1N2∑x=−N/2N/2∑y=−N/2N/2f(x,y)exp[−j⋅2π(ux+vy/N)]

Step 2: Resample the Fourier spectrum for each scale j and orientation l: *F*[*u*, *v-u tanθ_l_*].

Step 3: Apply a window function to partition the spectrum into wedge-shaped subbands.

Step 4: Wrap each wedge-shaped region into a rectangular form centered at the origin.

Step 5: Perform inverse Fourier transform to obtain curvelet coefficients:

(8) $$c^{D}(j,l,k)=\sum_{0\leq u,v<n}F[u,v]\cdot \phi_{j,l,k}^{D}[u,v]$$

This wrapping process allows localized frequency components to be reconstructed efficiently in the spatial domain.

#### 3.3.2. Approximation and Detail Images in Curvelet Domain

**Figure 4:**
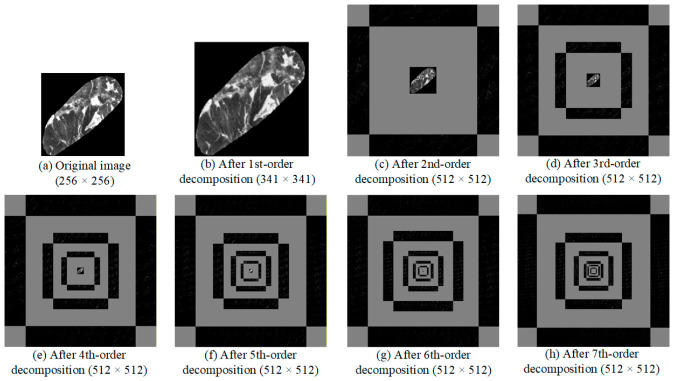
Curvelet-domain frequency representations at different decomposition scales (1–7), showing the change in spectral size and structure.

The curvelet transform decomposes an image in the frequency domain into one low-frequency approximation component and multiple high-frequency detail components. The approximation image is located at the center of the curvelet domain, representing the global intensity information, while the surrounding outer rings correspond to detail images that capture directional and high-frequency texture features. As the decomposition level increases, the curvelet transform captures progressively richer structural and directional variations. As illustrated in Figure 4, the frequency-domain representations from the 1st to the 7th scales demonstrate increasing complexity in the decomposition structure. Moreover, a higher number of detail subbands results in finer directional resolution.

In this study, a 5-scale curvelet transform is adopted for steak image analysis. The decomposition consists of 1 approximation image (central low-frequency component), 16 detail images (1st ring), 32 detail images (2nd ring), 32 detail images (3rd ring), and 64 detail images (outermost ring). This results in a total of 145 subband images in the curvelet domain. Each subband encodes specific spatial-frequency characteristics of the original image. Consequently, removing or modifying any detail subband leads to observable changes in the reconstructed spatial-domain image. Therefore, understanding the spatial and frequency localization of each subband enables targeted enhancement or suppression of specific features, which is essential for effective fat texture analysis.

#### 3.3.3. Effect of Angular Partitioning in Curvelet Domain

**Figure 5:**
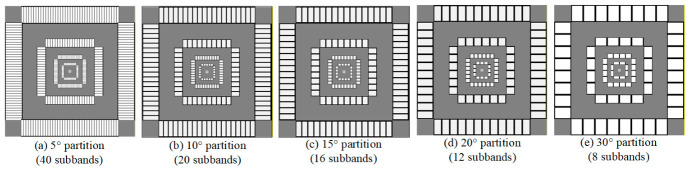
Illustration of different angular partitioning strategies in the curvelet domain and the corresponding numbers of directional subbands.

After applying the curvelet transform, the original image is decomposed into multiple detail subbands at different orientations. To analyze the relationship between detail subbands and angular partitioning, Figure 5 illustrates the partitioning scheme in the curvelet domain. The center of the curvelet domain corresponds to the approximation image, while each surrounding ring represents decomposed detail images at different scales and orientations. A smaller angular partition results in a larger number of directional subbands, enabling finer decomposition of fat marbling features. Conversely, larger partition angles produce fewer subbands and coarser feature representation. As illustrated in Figure 5e, the minimum number of directional subbands in the first ring is eight, and the total number of subbands must be divisible by four to maintain symmetry in the frequency domain. Therefore, the number of subbands *i* can be expressed as: *i* = 4*n*, where *n* is the smallest integer satisfying the divisibility constraint.

Based on experimental evaluation, a 15° angular partition, corresponding to 16 directional subbands, provides the best balance between feature resolution and computational efficiency. This configuration enables effective representation of fat texture patterns while avoiding unnecessary complexity.

#### 3.3.4. Square-Ring Filtering in Curvelet Domain

In this study, the curvelet transform combined with square-ring filtering is used to enhance fat texture and suppress background patterns for fat detection. In the curvelet domain, the central region corresponds to the low-frequency approximation image, which must be preserved during filtering. The surrounding rings represent detail subbands at different scales. Subbands closer to the center contain more global structural information but have lower directional resolution, whereas outer rings capture finer texture details and are more sensitive to noise [24]. Fat marbling features are distributed across specific detail subbands. Therefore, selecting the appropriate filtering ring is critical for enhancing fat-related features while suppressing irrelevant background textures. If the selected ring does not match the frequency range where fat features are prominent, the resulting segmentation performance will be suboptimal.

**Figure 6:**
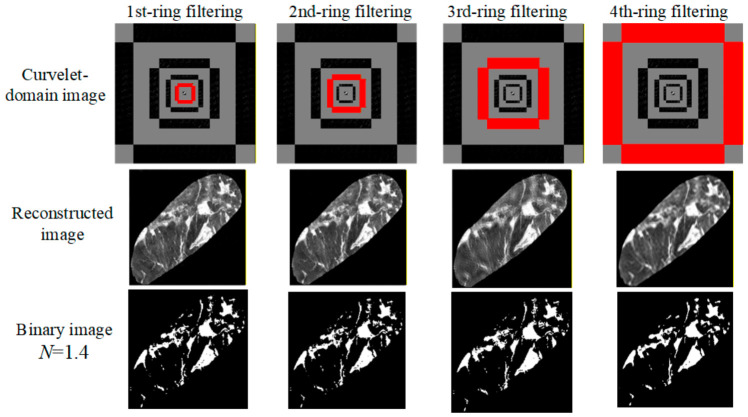
Comparison of square-ring filtering results using different curvelet-domain rings (highlighted in red). The fourth ring provides the optimal fat segmentation performance.

To determine the optimal configuration, the same test image is processed using different square-ring selections after applying a 5-scale curvelet transform, as shown in Figure 6. The results indicate that selecting the fourth ring yields the most effective fat segmentation, clearly enhancing fat structures while minimizing background interference. In contrast, using inner rings results in insufficient detail enhancement, whereas outer rings introduce excessive noise, degrading segmentation quality. These results confirm that the curvelet transform provides a flexible multi-scale and multi-directional framework, enabling selective enhancement of fat-related structures while suppressing irrelevant background textures, which is critical for reliable segmentation in complex meat images. Comparison of fat segmentation results obtained using square-ring filtering at different curvelet-domain rings. The selected ring (highlighted in red) determines the frequency band used for feature enhancement.

### 3.4. Separation of Lean Meat and Fat Regions

**Figure 7:**
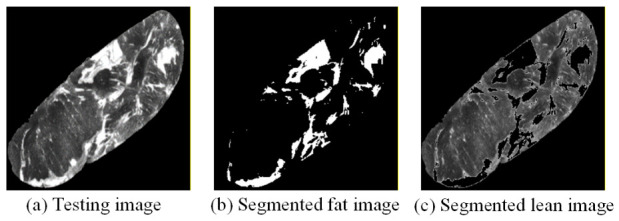
Segmentation results of the test image.

After filtering and reconstruction, the contrast between fat regions and the background is significantly enhanced, enabling effective separation using a binarization approach. In this study, a statistical interval estimation method is adopted to determine the threshold *T* for binarization, defined as:

(9) T=μ + Nσ

where *μ* and *σ* represent the mean and standard deviation of the grayscale intensity in the reconstructed image, respectively, and N denotes the scaling factor controlling the threshold sensitivity. Following the inverse curvelet transform, background texture components are largely suppressed, while fat structures are emphasized. This enhancement allows a simple thresholding operation to effectively separate fat regions from the surrounding tissue. The remaining regions are subsequently classified as lean meat. Figure 7 illustrates the segmentation results, including the original test image, the extracted fat regions, and the corresponding lean meat regions.

### 3.5. Uniformity Analysis of Fat Distribution

To further evaluate whether fat marbling exhibits aggregation or clustering defects, this study proposes a local projection method for analyzing fat distribution uniformity. The steak is first partitioned into several regions along its main axis, and the fat distribution within each region is projected and analyzed. A chi-square goodness-of-fit test is then applied to determine whether the fat distribution in each region follows a uniform pattern. If fat accumulation occurs, the distribution deviates from uniformity, indicating a potential defect in marbling structure.

#### 3.5.1. Convex Hull Detection of Steak Shape

**Figure 8:**
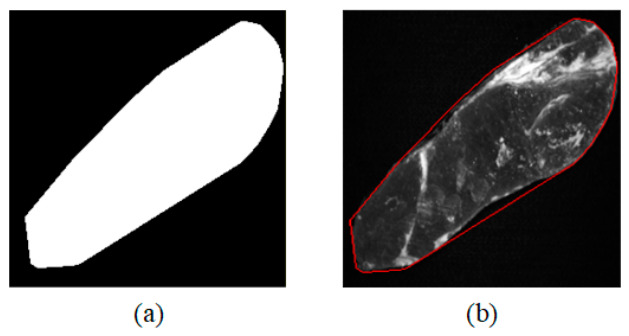
Convex hull representation of the steak region in the test image: (**a**) convex hull mask of the steak; (**b**) overlay of the convex hull on the original steak image.

To accurately determine the main axis of the steak, the convex hull of the steak region is first computed. This step eliminates irregular concave boundaries and prevents the generation of complex skeleton structures during subsequent processing. The convex hull is a fundamental concept in computational geometry, defined as the smallest convex polygon enclosing a set of points. In image processing, it is commonly used to fill gaps and simplify object contours. In this study, the convex hull is used to approximate the steak shape, thereby reducing boundary irregularities and facilitating reliable skeleton extraction for main-axis estimation. Figure 8a shows the convex hull of the steak region, while Figure 8b presents the overlay of the convex hull on the original steak image.

#### 3.5.2. Skeleton and Main Axis Detection of the Steak

**Figure 9:**
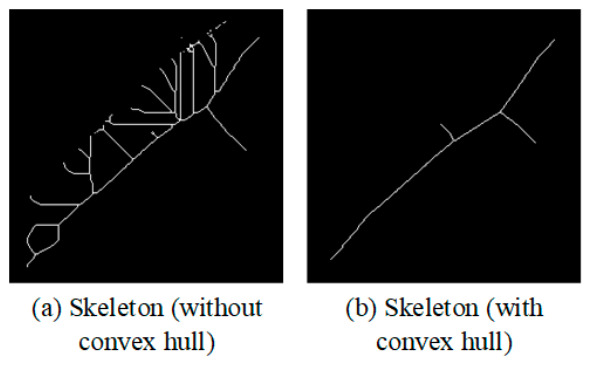
Skeletonization comparison without and with convex hull preprocessing, highlighting the reduction in spurious branches.

To analyze fat distribution uniformity, the steak is partitioned along its main axis, which is extracted from the object skeleton. The skeleton provides a compact representation of shape with the following properties: (1) one-pixel width; (2) located at the geometric center of the object; and (3) preservation of topological structure. To improve robustness, the skeleton is computed from the convex hull rather than directly from the steak region. As shown in Figure 9, skeletonization without convex hull preprocessing produces excessive spurious branches due to boundary irregularities, whereas convex hull-based processing yields a cleaner and more stable skeleton.

**Figure 10:**
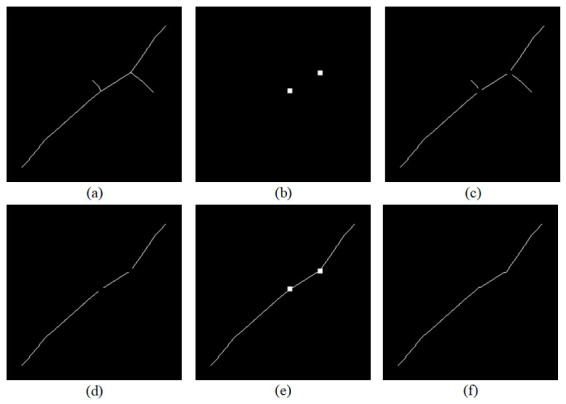
Procedure for removing spurious branches from the skeleton: (**a**) initial skeleton image; (**b**) detection of junction points; (**c**) removal of junction points and labeling of branches; (**d**) deletion of spurious branches; (**e**) merging of junction points with the refined skeleton; (**f**) final thinned skeleton representing the main axis.

To extract the main axis of the steak, the convex hull of the steak region is first computed, followed by skeletonization of the convex hull. The resulting skeleton is then pruned to remove spurious branches, yielding a clean representation of the main axis. Figure 10 illustrates the process of skeleton extraction and branch removal based on the convex hull. The procedure for main axis extraction is summarized as follows. Step 1: Compute the skeleton from the convex hull image (Figure 10a). Step 2: Detect junction points between the skeleton and spurious branches, and enhance them using dilation (Figure 10b). Step 3: Remove the junction points from the skeleton and label the corresponding branches (Figure 10c). Step 4: Identify and remove the labeled spurious branches (Figure 10d). Step 5: Merge the junction points with the pruned skeleton (Figure 10e). Step 6: Apply thinning to obtain a refined one-pixel-wide skeleton, representing the main axis of the steak (Figure 10f). This procedure effectively suppresses boundary-induced artifacts and preserves the dominant geometric structure, enabling robust and stable extraction of the steak’s main axis for subsequent regional analysis.

**Figure 11:**
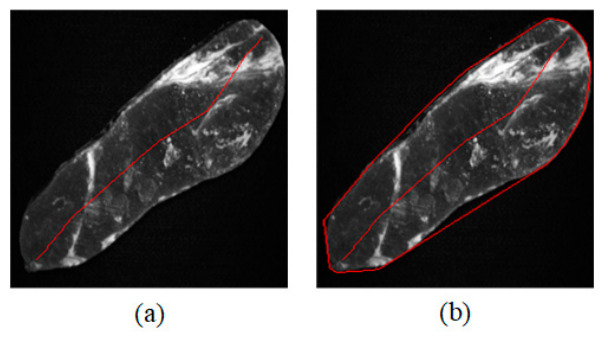
Extraction of the main axis and its overlay on the steak image: (**a**) overlay of the main axis on the steak image; (**b**) combined visualization of the convex hull and main axis overlaid on the steak.

After removing spurious branches from the skeleton, the main axis of the steak can be obtained. The convex hull and the extracted main axis are then overlaid onto the original steak image for visualization and subsequent analysis. As shown in Figure 10a, the skeleton derived from the convex hull is first obtained. Figure 10f presents the refined main axis after branch removal. Figure 11a illustrates the overlay of the main axis on the steak image, while Figure 11b shows the combined visualization of both the convex hull and the main axis superimposed on the steak.

#### 3.5.3. Main-Axis-Based Region Segmentation and Fat Distribution Projection

**Figure 12:**
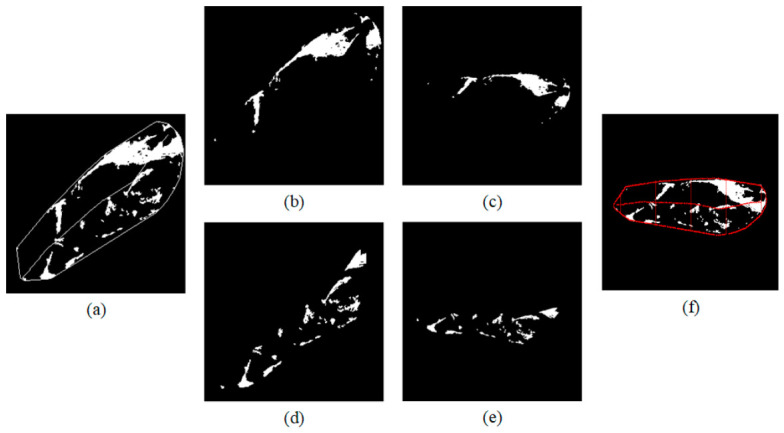
Region segmentation of the fat image based on the main axis: (**a**) fat image overlaid with convex hull and main axis; (**b**) segmented upper fat image; (**c**) rotated upper fat image (aligned with the horizontal main axis); (**d**) segmented lower fat image; (**e**) rotated lower fat image (aligned with the horizontal main axis); (**f**) final region segmentation of the fat image into eight subregions based on the main axis.

To more accurately evaluate fat concentration and aggregation in steak images, this study partitions the steak into multiple regions based on its main axis. Because fat accumulation defects may vary in size, the steak is divided into upper and lower halves along the main axis, and each half is further subdivided into four regions. First, the fat image is split into upper and lower halves along the main axis. Each half is then rotated to align the main axis horizontally, producing normalized upper and lower images. These aligned images are then divided into four equal segments along the main axis, yielding a total of eight regions. The segmentation process and the overlay of these regions on the fat image are illustrated in Figure 12.

**Figure 13:**
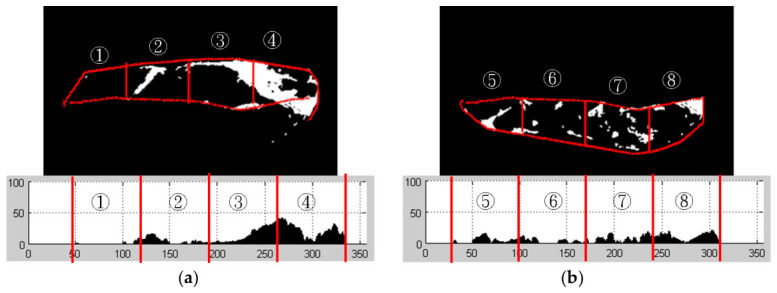
Projection-based distribution of fat patterns along the main axis: (**a**) upper-half regions (Regions 1–4) with corresponding projection profiles; (**b**) lower-half regions (Regions 5–8) with corresponding projection profiles.

If the regions are too large, excessive aggregation of fat features may lead to incorrect classification as non-uniform distribution; conversely, if the regions are too small, localized fat clusters may dominate individual regions, leading to false interpretation as uniform distribution. Based on empirical evaluation, dividing the steak into eight regions (four upper and four lower) provides a suitable balance. The upper regions are indexed from left to right as Regions 1–4, while the lower regions are indexed as Regions 5–8. For each region, fat distribution is projected onto a one-dimensional profile, and the resulting data are analyzed using a chi-square goodness-of-fit test to assess uniformity. The projection and regional distribution results are shown in Figure 13. This region-based projection strategy enables localized statistical analysis of fat distribution, improving sensitivity to clustering defects while maintaining robustness against global intensity variations.

#### 3.5.4. Chi-Square Goodness-of-Fit Test for Fat Distribution Uniformity

The spatial distribution of fat marbling is an important indicator of beef quality. While conventional grading methods often focus on the total amount of marbling, localized fat accumulation and distribution uniformity can also significantly influence the perceived quality of beef products. To objectively evaluate marbling uniformity, this study employs the chi-square goodness-of-fit test to determine whether the fat distribution within each steak region follows a uniform distribution.

After fat segmentation, the steak is divided into eight regions along its principal axis. For each region, the segmented fat pixels are projected onto the principal axis and grouped into *k* projection intervals. The observed frequency distribution is then compared with the expected frequency distribution under the assumption of uniform fat distribution [25]. The null and alternative hypotheses are defined as follows:

*H*_0_: The fat distribution within a region is uniform;

*H*_1_: The fat distribution within a region is non-uniform.

The chi-square test statistic is computed as:

(10) $$\chi^{2}=\sum_{i=1}^{k}\frac{{\left(E_{i}-O_{i}\right)}^{2}}{E_{i}},$$

where *O*_*i*_ denotes the observed frequency in the *i*-th bin, *E*_*i*_ represents the expected frequency under a uniform distribution, and *k* is the number of bins (i.e., projection intervals within each region). Under the assumption of a uniform distribution, the expected frequency is defined as:

(11) $$E_{i}=N/k,\;i\;=\;1,\;2,\;\ldots ,\;k,$$

where *N* is the total number of observations in the region. The degrees of freedom for the chi-square test are given by: df = *k* − 1. The computed test statistic χ^2^ is then compared against the chi-square distribution with (*k* − 1) degrees of freedom. Specifically, the corresponding *p*-value is obtained from the chi-square distribution table (or cumulative distribution function), which represents the probability of observing a test statistic at least as extreme as the calculated value under the null hypothesis.

To ensure statistical validity, two empirical rules are applied before hypothesis testing [25]. First, when an observed frequency *X*_*i*_ < 5, adjacent bins are merged until the condition *X*_*i*_ ≥ 5 is satisfied. Second, if ten consecutive observations satisfy *X*_*i*_ > 15, the region is directly classified as a fat accumulation region without further statistical testing. These preprocessing steps improve the robustness of the chi-square test and prevent misleading conclusions due to sparse or highly skewed data. Finally, the computed *p*-value is compared with the significance level *α* = 0.05. If *p* < 0.05, the null hypothesis is rejected, indicating non-uniform fat distribution (i.e., fat accumulation). Otherwise, the region is considered to exhibit a uniform distribution. The incorporation of degrees of freedom and statistical significance testing ensures that the proposed method provides a rigorous and quantitative evaluation of fat distribution uniformity, enhancing both interpretability and reliability in practical inspection scenarios.

**Figure 14:**
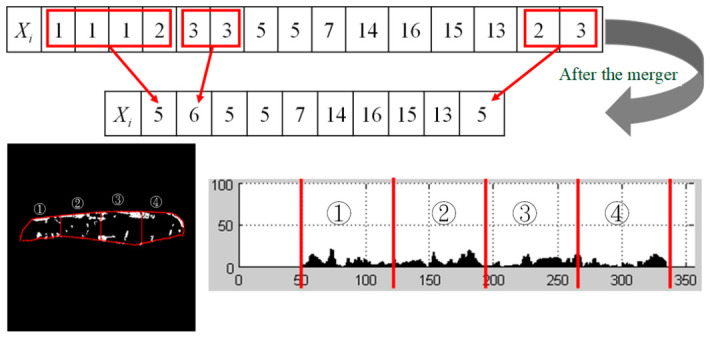
Illustration of the preprocessing step (Rule 1) for the chi-square goodness-of-fit test. Observed frequencies below 5 are iteratively merged with adjacent bins to meet the minimum frequency requirement, ensuring the validity of the statistical test. Region 2 is shown as an example.

**Figure 15:**
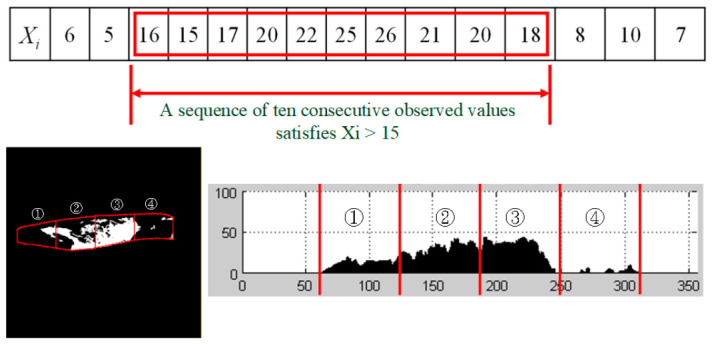
Illustration of Rule 2 for detecting fat accumulation. A region is classified as non-uniform when ten consecutive observed values exceed the threshold (*X_i_* > 15), indicating localized clustering. Region 2 is shown as an example.

The effectiveness of the proposed fat distribution analysis is quantitatively validated using the chi-square goodness-of-fit test applied to projection profiles of segmented regions. As illustrated in Figure 14, Rule 1 ensures statistical reliability by merging adjacent bins when the observed frequency *X_i_* is <5, thereby preventing instability due to sparse data. In addition, Rule 2 (Figure 15) identifies fat accumulation regions when ten consecutive observations satisfy *X_i_* > 15, allowing early detection of pronounced clustering without further statistical computation. After preprocessing, the chi-square test statistic is computed as the sum of squared deviations between observed and expected frequencies, assuming a uniform distribution, with degrees of freedom *k* − 1. The resulting *p*-values are then obtained from the chi-square distribution and compared against the significance level α = 0.05 to determine distribution uniformity.

The use of the chi-square goodness-of-fit test provides two important advantages. First, it converts subjective visual assessment of marbling distribution into an objective statistical evaluation. Second, it enables localized analysis of fat accumulation rather than relying solely on overall fat content. This allows regions exhibiting excessive fat concentration to be identified and quantified, thereby providing additional information for quality grading.

**Figure 16:**
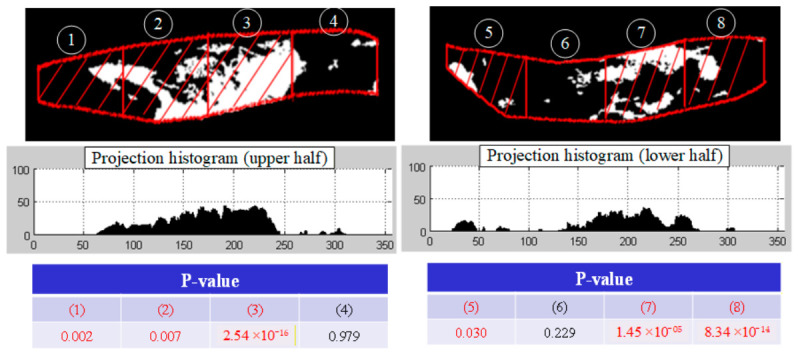
Example of non-uniform fat distribution. Regions with *p*-value < 0.05 indicate significant deviation from uniformity, corresponding to fat accumulation, except regions 4 and 6.

**Figure 17:**
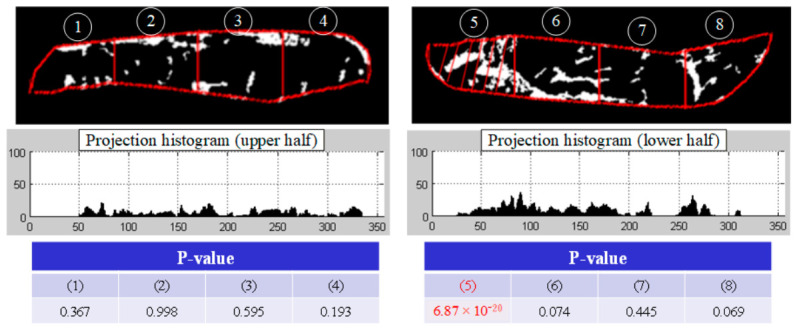
Example of uniform fat distribution. Regions with *p*-value ≥ 0.05 indicate no significant deviation from uniformity except region 5.

**Figure 18:**
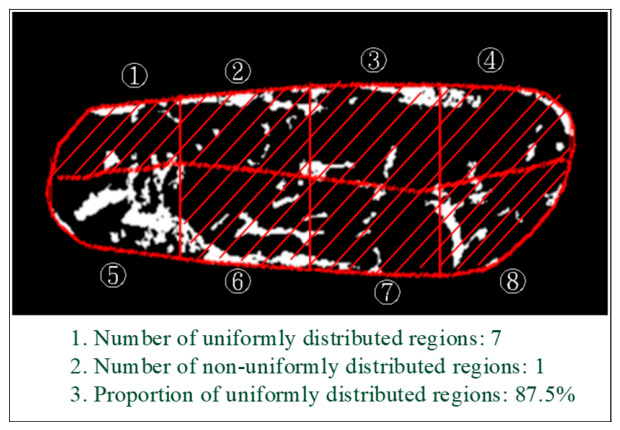
Final uniformity analysis of fat distribution using the chi-square goodness-of-fit test. Regions are classified as uniform or non-uniform, with an overall uniformity ratio of 87.5% in the illustrated example.

To validate the effectiveness of the proposed statistical analysis, the detected non-uniform regions were compared with visual observations obtained from manual inspection. Figure 16 and Figure 17 present representative examples of non-uniform and uniform fat distributions, respectively. In Figure 16, several regions have *p*-values < 0.05, indicating statistically significant deviations from uniformity and revealing localized fat accumulation. In contrast, most regions in Figure 17 have *p*-values ≥ 0.05, indicating a more homogeneous fat distribution. Building on these regional decisions, Figure 18 summarizes the final uniformity assessment, classifying each segmented region as uniform or non-uniform. Regions with *p* < 0.05 are classified as non-uniform (fat accumulation). In this example, seven regions are uniform, and one is non-uniform, yielding a uniformity ratio of 87.5% and demonstrating effective detection of localized fat clustering. The observed agreement between statistically detected non-uniform regions and visually apparent fat accumulation areas supports the validity of the proposed chi-square-based uniformity analysis and demonstrates its consistency with human inspection.

### 3.6. Support Vector Machines Applied to Color Classification of Lean Meat Regions

In this study, the fat regions are analyzed to evaluate the uniformity of marbling distribution, while the lean regions are used to extract color feature vectors, including the mean and standard deviation of each component in the color space. These features are then input into a Support Vector Machine (SVM) model to assess the redness of the lean meat. After obtaining the lean redness grade, the overall quality grading of the steak is performed by integrating the fat distribution characteristics across multiple regions with the lean redness ratios. Appropriate weighting factors are assigned to these ratios to enable a comprehensive and reliable determination of beef quality.

**Figure 19:**
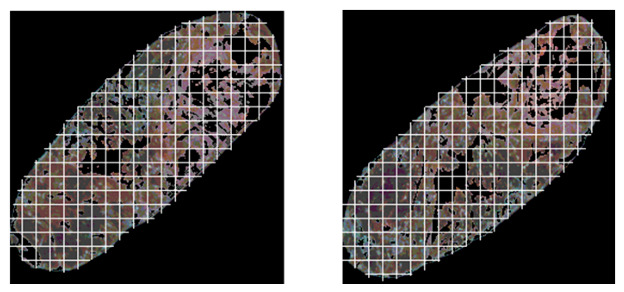
Grid-based partitioning of lean meat regions for front and back sides of the sample image (13 × 13 pixels per grid; 19 × 19 grids per image).

In this study, each image is first partitioned into 13 × 13-pixel grids (Figure 19), yielding 361 (19 × 19) subregions per image. If more than half of a grid is background, it is discarded and excluded from both training and testing. Color features are then extracted from each remaining grid. The RGB components are suitable for describing color variation on beef surfaces [26]. After feature extraction, the data are normalized and used as input to a Support Vector Machine (SVM) for training. Because SVM is a supervised learning method, an effective classification model must be built using labeled training data. Subsequently, the trained model is applied to classify test samples. In this study, an SVM-based learning scheme for lean meat color evaluation is first established, and an effective classification model is constructed using training data.

**Figure 20:**
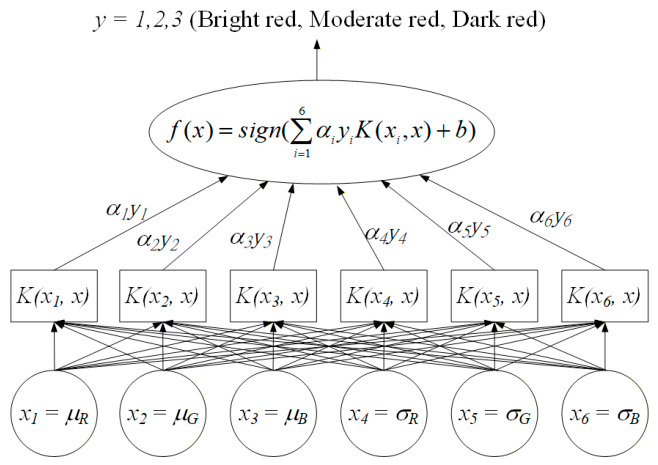
Schematic diagram of the Support Vector Machine (SVM) model used in this study.

Figure 20 shows the architecture of the SVM-based classification model for lean-meat color analysis. Statistical color features (mean and standard deviation of RGB channels) are used as input variables. The decision function is constructed from kernel evaluations between the input vector and support vectors, enabling multi-class classification of meat redness levels. The corresponding output labels, *y_i_*, were defined as 1 (bright red), 2 (moderate red), and 3 (dark red).

### 3.7. Quality Grading System Integrating Fat Marbling Distribution and Lean Meat Color Variation

**Figure 21:**
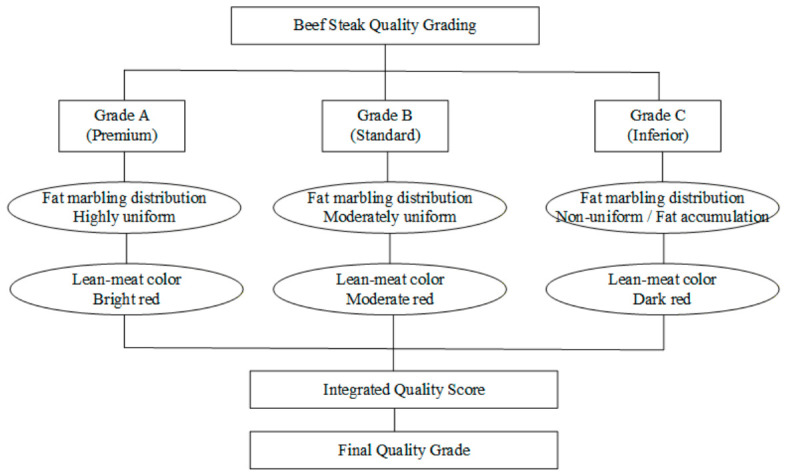
Beef steak quality grading criteria based on fat marbling distribution and lean-meat color.

Unlike conventional grading methods that rely primarily on marbling quantity, the proposed framework jointly evaluates marbling distribution uniformity and lean-meat color characteristics. Figure 21 illustrates the grading criteria adopted in this study. Consequently, steaks exhibiting evenly distributed marbling and bright-red lean meat are assigned higher quality grades, whereas steaks containing localized fat accumulation or darker lean-meat color receive lower grades. This strategy provides a more comprehensive and interpretable assessment of beef quality.

**Figure 22:**
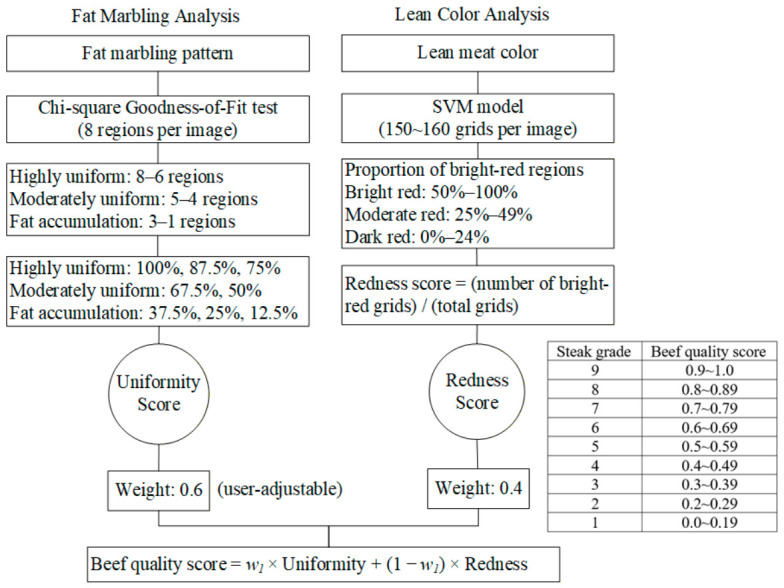
Quality evaluation workflow for test samples based on fat distribution and lean color analysis.

To achieve a comprehensive and practical assessment of beef quality, the proposed framework integrates fat marbling distribution and lean meat color variation into a unified grading system. As illustrated in Figure 22, the framework consists of two parallel analysis branches. Fat marbling uniformity is evaluated using a chi-square goodness-of-fit test across eight regions, producing a uniformity score, while lean meat color is assessed using an SVM-based model that computes a redness score from grid-level color features. These two complementary attributes are then fused through a weighted scheme to generate a final beef quality score, which is mapped to discrete grading levels for interpretable evaluation.

**Figure 23:**
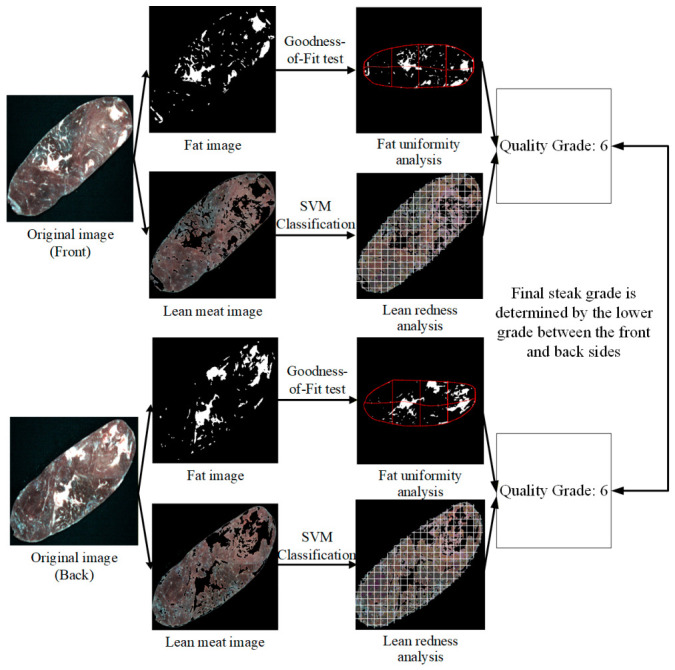
Overall grading framework combining fat distribution uniformity and lean color analysis for front and back surfaces.

Table 2 and Figure 23 present both quantitative and visual analyses of the same steak sample for cross-validation. Table 2 reports fat uniformity and lean redness for both front and back surfaces, while Figure 23 illustrates the corresponding segmentation and spatial distribution patterns. The results show strong consistency between statistical measurements and visual observations: regions identified as non-uniform (*p* < 0.05) correspond to visible fat accumulation, and higher redness scores align with brighter lean color. Additionally, the use of a conservative grading strategy—selecting the lower score between front and back surfaces—enhances robustness. Overall, the framework provides a statistically reliable, visually consistent, and practically applicable solution for automated beef quality grading.

## 4. Experiments, Results, and Discussion

To validate the feasibility of the proposed method, this study conducts system implementation, experimental validation, and performance evaluation. The experimental results are used to assess whether the proposed approach achieves the expected performance in defect detection and quality grading. Furthermore, the proposed method is compared with other detection and classification approaches to demonstrate its effectiveness.

### 4.1. Configuration of Quality Grading System

The experimental setup included a personal computer (Intel Core i7-6500U CPU, 4 GB RAM, Windows 10), a 5 MP color CCD camera, a 13–130 mm zoom lens, and ring-shaped illumination. All images were resized to 256 × 256 pixels and processed using MATLAB R2013b. The proposed system integrates homomorphic filtering for frost suppression, curvelet-based segmentation for separating fat and lean regions, and subsequent analysis of fat distribution uniformity and lean meat redness. Based on these features, the system outputs both performance metrics and the final steak quality grade.

### 4.2. Performance Metrics for Beef Fat Detection

In this study, the performance of the proposed beef fat detection method is evaluated using Type I error (α) and Type II error (β), which are commonly used statistical measures for assessing detection accuracy.

1. Lean meat misclassification rate (α)

The Type I error (α) is defined as the proportion of pixels in the lean meat region that are incorrectly classified as fat pixels in the detection results. This metric represents the lean meat misclassification rate and can be expressed as follows:

(12) $$(\alpha )\%\;=\;\frac{\mathrm{Area}\;\mathrm{of}\;\mathrm{lean}\;\mathrm{meat}\;\mathrm{misclassified}\;\mathrm{as}\;\mathrm{fat}}{\mathrm{Actual}\;\mathrm{lean}\;\mathrm{meat}\;\mathrm{area}}\times 100\%$$

2. Fat detection rate (1 − β)

The Type II error (β) is defined as the proportion of fat pixels that are incorrectly classified as lean meat pixels in the detection results. Accordingly, the fat detection rate is represented by (1 − β), which indicates the proportion of fat area correctly detected. This can be expressed as follows:

(13) $$(1\;-\;\beta )\%\;=\;\frac{\mathrm{Correctly}\;\mathrm{detected}\;\mathrm{fat}\;\mathrm{area}}{\mathrm{Actual}\;\mathrm{fat}\;\mathrm{area}}\times 100\%$$

3. Correct classification rate (CR)

The correct classification rate (CR) is defined as the ratio of the correctly classified lean meat area plus the correctly classified fat area to the total steak area in the image. This metric reflects the overall segmentation accuracy of the proposed method and is given by:

(14) $$(\mathrm{CR})\%\;=\;\frac{\mathrm{Correctly}\;\mathrm{classified}\;\mathrm{lean}\;\mathrm{meat}\;\mathrm{area}\;+\;\mathrm{Correctly}\;\mathrm{classified}\;\mathrm{fat}\;\mathrm{area}}{\mathrm{Total}\;\mathrm{steak}\;\mathrm{area}}\times 100\%$$

### 4.3. Optimal Parameter Settings for the Proposed Methods

In this study, key parameters of the proposed methods were systematically optimized, including the high-frequency gain of the homomorphic filter, the decomposition level of the curvelet transform, the segmentation threshold for the fat region separation, the steak partition size for fat uniformity analysis, and parameter settings of the SVM Model for lean-meat color analysis. The optimal parameter combination was determined through performance evaluation.

#### 4.3.1. Selection of the High-Frequency Range for Homomorphic Filtering

Homomorphic filtering was employed to suppress illumination effects and enhance the reflectance components associated with frost on the steak surface. In this study, the low-frequency component was fixed (0.5) to preserve the overall illumination characteristics of the image, and only the high-frequency range *h* was varied to reduce frost-induced reflections while preserving fat texture features.

**Figure 24:**
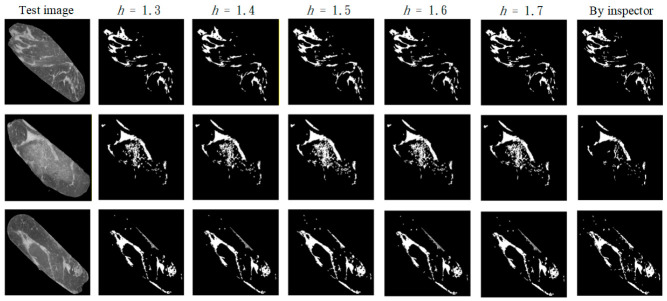
Partial fat segmentation results under different high-frequency ranges of homomorphic filtering.

To determine the optimal setting, *h* was evaluated at 1.3, 1.4, 1.5, 1.6, and 1.7, with the binarization parameter *N* = 1.75. The corresponding segmentation results are shown in Figure 24, and quantitative performance is summarized in Table 3. The results indicate that the high-frequency range significantly affects the trade-off between fat detection and lean meat misclassification. Lower values (*h* = 1.3, 1.4) yield lower misclassification rates (*α* = 3.54% and 3.77%) but also reduce detection performance (88.14% and 88.80%). Increasing *h* to 1.5 improves the detection rate to 92.68%, the highest among all settings, while maintaining an acceptable misclassification rate (*α* = 4.96%) and a high overall accuracy (*CR* = 94.09%).

Further increasing *h* to 1.6 and 1.7 does not improve performance, as detection rates decrease to 89.52% and 89.60%, respectively. Considering that accurate identification of fat regions is critical for subsequent marbling analysis, a slightly higher α is acceptable if detection performance improves significantly. Therefore, *h* = 1.5 is selected as the optimal high-frequency range, providing the best balance between sensitivity and specificity under frost-interference conditions.

#### 4.3.2. Selection of the Curvelet Transform Decomposition Level

The decomposition level of the curvelet transform controls the representation of directional details and structural features in the image. While increasing the decomposition level enhances the ability to capture fine textures, excessively high levels do not necessarily improve fat detection performance. Therefore, different decomposition levels were evaluated to determine an optimal balance between fat detection rate (1 − β) and lean meat misclassification rate (α).

**Figure 25:**
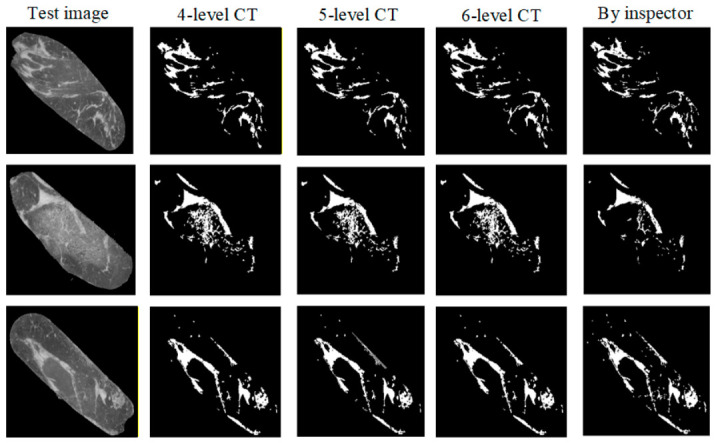
Partial fat segmentation results obtained using different curvelet transform decomposition levels.

In this experiment, the binarization parameter in the binarization threshold (*μ* ± *Nσ*) was fixed at *N* = 1.75, and curvelet decomposition levels from 3 to 6 were tested. Higher levels (≥7) failed to detect fat regions. The segmentation results are shown in Figure 25, and quantitative performance is summarized in Table 4. As indicated, a 3-level decomposition yields a relatively low misclassification rate (*α* = 4.43%) but a limited detection rate (91.06%), suggesting incomplete preservation of fat regions. Increasing the level to 4 improves detection (92.06%) but increases misclassification (*α* = 5.43%). The 5-level decomposition achieves the highest detection rate (92.68%) with a moderate misclassification rate (*α* = 4.97%), providing the best overall balance. Although its overall classification rate (*CR* = 94.09%) is slightly lower than that of other levels (94.42%), the difference is marginal.

At 6 levels, performance deteriorates, with a reduced detection rate (91.06%) and increased misclassification, indicating no benefit from further decomposition. Considering the importance of accurately preserving fat regions for subsequent marbling analysis, a 5-level curvelet transform is selected as the optimal setting.

#### 4.3.3. Selection of the Binarization Threshold for the Reconstructed Image

Following filtering, binarization was applied using the statistical threshold (*μ* ± *Nσ*) to separate fat regions from the background. The parameter *N* was optimized to balance fat detection (1 − β) and lean meat misclassification (*α*), while fixing the homomorphic filter at *h* = 1.5 and the curvelet decomposition level at 5.

**Figure 26:**
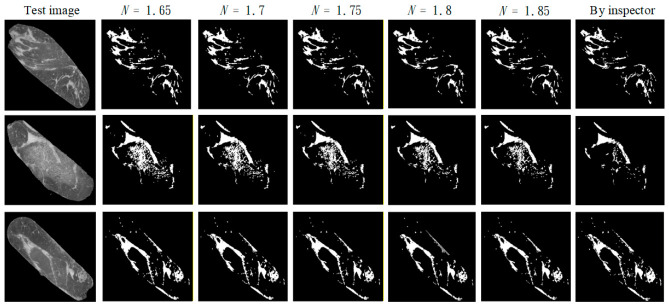
Segmentation results obtained using different binarization threshold values *N*.

The segmentation results for different *N* values are shown in Figure 26, with quantitative performance summarized in Table 5. The results indicate that N strongly influences the trade-off between sensitivity and specificity. A lower threshold (*N* = 1.65) achieves the highest detection rate (94.63%) but also produces the highest misclassification rate (*α* = 6.31%) and lowest *CR* (92.23%), indicating over-segmentation. As *N* increases to 1.70 and 1.75, misclassification decreases (5.60% and 4.97%), while detection remains high (93.72% and 92.68%). Notably, *N* = 1.75 provides the most balanced performance (*CR* = 94.09%).

Although *N* = 1.85 yields the lowest misclassification rate (3.91%) and highest *CR* (94.63%), its detection rate drops significantly (90.29%), indicating under-segmentation. Since accurate preservation of fat regions is critical for subsequent marbling analysis, *N* = 1.75 is selected as the optimal threshold, achieving the best balance between detection accuracy and robustness.

#### 4.3.4. Selection of the Steak Partition Size for Fat Uniformity Analysis

Following fat segmentation, the steak image is divided into multiple regions to evaluate fat distribution uniformity. The partition size directly affects analysis accuracy: overly large regions (few partitions) may merge localized variations and falsely indicate non-uniformity, whereas overly small regions (many partitions) may fragment continuous fat areas and incorrectly suggest uniformity.

**Figure 27:**
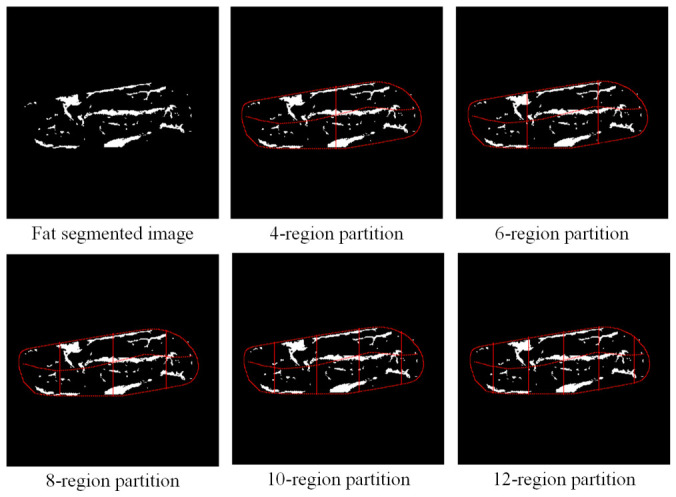
Schematic illustration of different numbers of partitioned regions for steak fat uniformity analysis.

Figure 27 illustrates different partition configurations, and the corresponding results are summarized in Table 6. The results show that partition size significantly influences uniformity interpretation. With four regions, all areas are classified as non-uniform, indicating over-aggregation. Increasing to six regions yields a balanced outcome (three uniform, three non-uniform), corresponding to moderate uniformity. When divided into eight regions, six are identified as uniform and two as non-uniform, resulting in a “densely uniform” classification that matches manual inspection. However, further increasing the number of regions (10 or 12) leads to inconsistent results, as excessive partitioning fragments continuous fat structures and distorts spatial distribution patterns. Considering both agreement with manual inspection and the balance between over- and under-segmentation, eight regions are selected as the optimal partition setting for reliable fat uniformity analysis.

#### 4.3.5. Parameter Selection of the SVM Model for Lean-Meat Color Analysis

An SVM classifier with a radial basis function (RBF) kernel was employed for lean-meat color classification due to its effectiveness in handling nonlinear and high-dimensional data while requiring only two tunable hyperparameters, the cost parameter (C) and gamma parameter (γ). The input feature vector consisted of six RGB color descriptors, the mean values (*μ_R_*, *μ_G_*, *μ_B_*) and standard deviations (*σ_R_*, *σ_G_*, *σ_B_*) of the lean-meat regions. The normalized feature data were categorized into three classes: bright red, moderate red, and dark red. The corresponding SVM parameter settings are summarized in Table 7.

To ensure an unbiased evaluation, the SVM classifier was developed using independent training, validation, and testing datasets. The validation dataset was used exclusively for hyperparameter optimization and model selection, whereas the testing dataset was reserved for final performance evaluation. Consequently, the validation accuracy reported during parameter tuning and the testing accuracy reported for the final classifier correspond to different evaluation stages.

**Figure 28:**
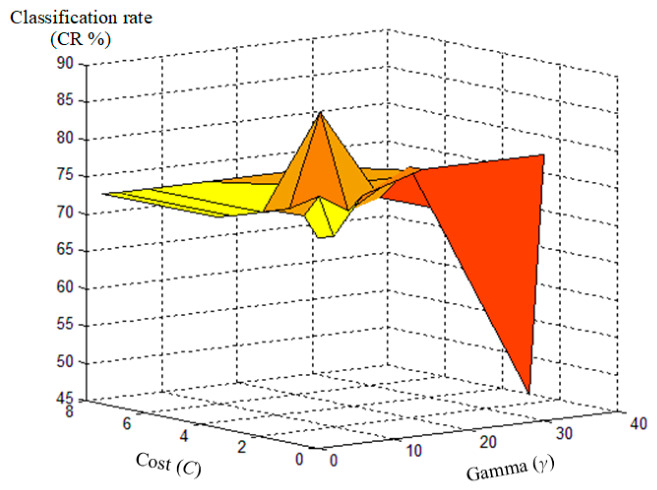
Classification accuracy (CR %) of the SVM model under different combinations of hyperparameters C and γ.

To identify the optimal configuration, 840 training and validation images were used to evaluate different combinations of *C* and *γ*. The best-performing parameter pair was found to be (*C*, *γ*) = (8, 2). To further investigate the effect of parameter variation on classification performance, additional experiments were conducted by adjusting C and γ over a predefined range. Specifically, C was varied from 2^1^ to 2^5^, and *γ* from 2^−1^ to 2^3^, as summarized in Table 8, with the corresponding results illustrated in Figure 28. The results indicate that classification performance is sensitive to hyperparameter selection, with the optimal combination achieving the highest accuracy (87.5%). Overall, the RBF-based SVM provides a robust and efficient solution for lean meat color classification.

Following hyperparameter optimization, the trained SVM model was evaluated on an independent test set to assess its classification performance. A total of 210 test images were processed, where classification was determined based on the dominant color category (bright red, moderate red, or dark red) identified from grid-level predictions.

The model achieved an overall accuracy of 96.67%, demonstrating strong agreement with human evaluation. As summarized in Table 9, the classifier correctly identified 74 bright red, 77 moderate red, and 59 dark red samples. Misclassifications are minimal and occur primarily between adjacent categories (bright red and moderate red), with only one instance involving the dark red class. This indicates that the proposed feature representation and SVM classifier effectively capture the discriminative characteristics of lean meat color.

Further quantitative evaluation (Table 10) shows consistently high performance across all classes, with F1-scores exceeding 95%. In particular, the dark red class achieves near-perfect performance (F1-score: 99.16%), reflecting strong separability in lower-quality samples. Minor confusion between the bright red and moderate red categories is expected due to their similar color distributions. The high macro-average F1-score (96.84%) and weighted F1-score (96.68%) confirm the robustness and reliability of the proposed method under practical conditions. Overall, the results demonstrate that the proposed SVM-based color analysis provides a robust, accurate, and interpretable solution for lean meat color grading, forming a reliable component of the overall beef quality evaluation framework.

Overall, the adopted parameter values were selected through systematic parameter optimization experiments rather than empirical observation alone. These experiments effectively serve as an ablation study, revealing the influence and contribution of each major component to the overall system performance. The results confirm that the chosen parameter configuration provides the optimal trade-off among fat detection capability, grading accuracy, and robustness, thereby enhancing the reliability, reproducibility, and practical applicability of the proposed beef quality grading framework.

### 4.4. Performance Comparison of Fat Segmentation and Quality Grading Methods

To comprehensively evaluate the effectiveness of the proposed framework, this section presents a two-stage performance comparison focusing on both fat segmentation and overall quality grading. Section 4.4.1 compares the proposed method with conventional spatial- and frequency-domain approaches to assess its capability in accurately extracting fat marbling regions under challenging conditions. Section 4.4.2 evaluates the performance of direct deep learning models for end-to-end beef quality grading and contrasts them with the proposed feature-driven approach, highlighting the advantages of integrating domain knowledge for improved accuracy and reliability.

#### 4.4.1. Performance Comparison of Fat Segmentation Methods

**Figure 29:**
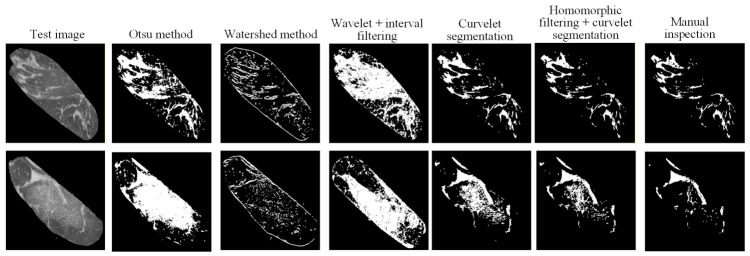
Comparison of fat marbling detection results for selected steak sample images using different methods.

Figure 29 illustrates a qualitative comparison of fat marbling segmentation results obtained using representative spatial- and frequency-domain methods, including Otsu thresholding [27], watershed segmentation [28], wavelet transform with interval filtering [29], curvelet segmentation, and the proposed homomorphic filtering combined with curvelet segmentation. As shown, the watershed method exhibits limited capability in separating fat regions from the background, leading to noticeable missed detections of major fat structures. Although the Otsu and wavelet-based methods are able to detect a large portion of fat regions, they suffer from substantial false positives, primarily due to the misclassification of frost-contaminated lean meat as fat. In contrast, curvelet-based approaches demonstrate improved structural representation, yielding segmentation results that more closely resemble manual inspection. However, the curvelet-only method remains sensitive to frost-induced artifacts, resulting in residual misclassification. By incorporating homomorphic filtering, the proposed method effectively suppresses illumination variations and frost interference, producing cleaner and more reliable fat segmentation outcomes.

The quantitative results in Table 11 further corroborate these observations. The proposed method achieves a detection rate (1 − β) of 92.68%, a false positive rate (α) of 4.97%, and a correct classification rate (CR) of 94.09%, indicating a well-balanced trade-off between sensitivity and specificity. While the Otsu method attains the highest detection rate (99.77%), it also exhibits a significantly high false positive rate (41.24%), reflecting severe over-segmentation. Similarly, the wavelet-based method yields a relatively high detection rate (95.24%) but suffers from the highest false positive rate (63.77%) and the lowest CR (43.14%), limiting its practical applicability. The watershed method reduces false positives but at the expense of a substantially lower detection rate (71.49%), indicating insufficient sensitivity to true fat regions. The curvelet-only method achieves the lowest false positive rate (2.54%) and the highest CR (95.12%); however, its lower detection rate (84.11%) suggests a tendency to miss certain fat regions.

Overall, the proposed homomorphic filtering combined with curvelet segmentation demonstrates the most robust and balanced performance under frost-interference conditions, effectively reducing false detections while maintaining high sensitivity. These results confirm its superiority over conventional spatial- and frequency-domain methods for accurate and reliable fat marbling segmentation.

#### 4.4.2. Performance Comparison with Deep Learning Models for Direct Beef Quality Grading

To evaluate the effectiveness of deep learning approaches for direct beef quality grading, five representative models, including DenseNet121 [30], DenseNet201 [31], YOLOv8 [32], YOLOv11 [33], and YOLOv26 [34], were implemented and evaluated using a five-fold cross-validation protocol. To ensure a fair comparison, all deep learning models and the proposed method were evaluated using identical five-fold cross-validation partitions, and the reported results represent the mean and standard deviation obtained from the five folds. The average accuracy, precision, recall, and F1-score are summarized in Table 12.

The results indicate that the YOLO-based models generally achieved better grading performance than the DenseNet architectures. Among the evaluated deep learning models, YOLOv26 achieved the highest average accuracy of 75.43% and an F1-score of 56.35%, indicating a better balance between precision and recall. YOLOv8 also demonstrated competitive performance, achieving an accuracy of 74.03% and an F1-score of 55.27%. In contrast, DenseNet121 and DenseNet201 achieved accuracies of 62.65% and 63.99%, respectively, but had comparatively lower recall and F1-scores. These findings suggest that direct image-level grading remains challenging due to the complex visual characteristics of beef quality attributes, including marbling distribution and lean-meat color variation.

The proposed method achieved an average accuracy of 90.38% (±3.86%), precision of 89.98% (±4.57%), recall of 87.68% (±5.14%), and F1-score of 88.81% (±4.85%). Compared with the evaluated deep learning models, the proposed framework consistently produced higher average performance across all evaluation metrics while maintaining relatively low variation among the cross-validation folds. These improvements may be attributed to the incorporation of domain-specific information, including fat marbling distribution uniformity and lean-meat color characteristics, which are explicitly modeled through statistical analysis and machine-learning-based classification.

It should be noted that the objective of this comparison is not to suggest that conventional machine-learning approaches universally outperform deep learning methods. Rather, the results indicate that, for the investigated dataset and grading criteria, the integration of frequency-domain image enhancement, statistical marbling analysis, and lean-meat color evaluation provides a complementary and highly interpretable framework for beef quality assessment. The findings therefore highlight the potential benefits of combining domain knowledge with image analysis techniques when addressing specialized food-quality inspection tasks.

### 4.5. Dynamic Inspection Performance and Industrial Deployment Feasibility

**Figure 30:**
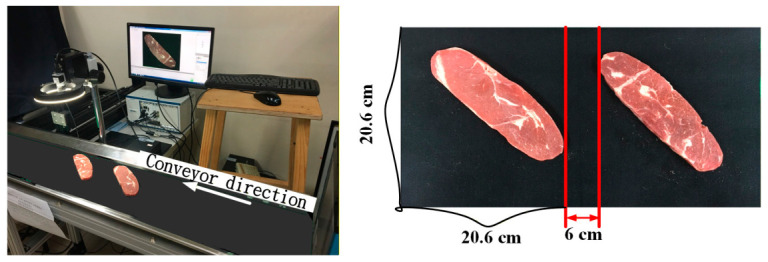
Experimental hardware setup for the proposed dynamic beef inspection system and the imaging field of view and sample spacing.

**Figure 31:**
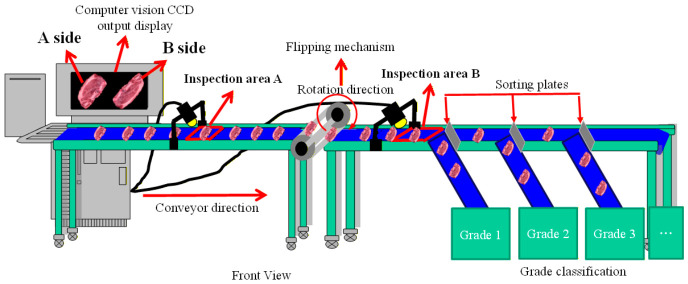
Front-view schematic of the proposed dynamic vision-based beef inspection and grading system. The system captures both A-side and B-side images using a dual-stage inspection process with a flipping mechanism, followed by automated classification.

To evaluate the practical applicability of the proposed framework in industrial environments, the static inspection system was extended to a conveyor-based dynamic inspection platform. Figure 30 shows the experimental hardware setup, which consists of a conveyor belt, a CCD camera, an illumination module, and a computer vision processing unit. Furthermore, an idea for a dual-sided inspection mechanism is proposed (Figure 31) to acquire both front-side (A-side) and back-side (B-side) images of each beef steak through an automated flipping process, enabling comprehensive quality evaluation during continuous transportation.

To evaluate the robustness of the proposed framework under dynamic inspection conditions, experiments were conducted at conveyor speeds ranging from 14.80 to 29.98 cm/s using the optimal parameters obtained from the static experiments. Table 13 summarizes the grading performance under different conveyor speeds. The proposed method achieved the highest accuracy of 89.45% at 14.80 cm/s (Speed 40) and maintained stable performance at moderate speeds 20.75–25.85 cm/s (Speed 50–60), with accuracies above 87.8% and F1-scores above 86%. As conveyor speed increased to 28.63–29.98 cm/s (Speed 70–80), grading performance gradually declined, reducing accuracy to 85.53–83.75% and F1-score to 83.93–82.31%. This performance degradation is primarily attributed to motion blur, which reduces the visibility of fine marbling textures and subtle lean-meat color variations required for accurate quality grading.

**Figure 32:**
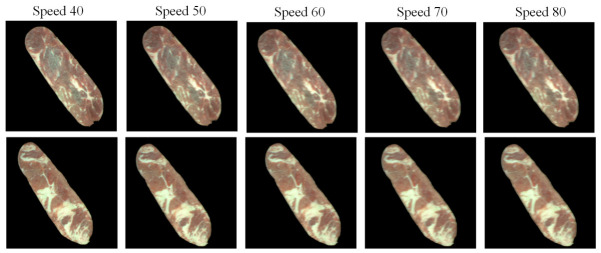
Sample images captured at different conveyor speeds in the dynamic inspection system. Increasing conveyor speed leads to noticeable motion blur, while the overall fat texture structure remains partially preserved.

Representative images captured at different conveyor speeds are shown in Figure 32. Although increasing conveyor speed causes progressive image blurring, the major marbling structures and overall color characteristics remain visually recognizable. This observation explains why the proposed framework continues to maintain acceptable grading performance even under relatively fast conveyor operation.

Overall, the dynamic experiments demonstrate that the proposed framework is robust under practical conveyor-based inspection conditions. In particular, the speed range of 14.80–25.85 cm/s (Speed 40–60) provides the best balance between inspection throughput and grading reliability, achieving accuracies above 87.8% and F1-scores above 86%. These results confirm the feasibility of deploying the proposed method in medium-speed industrial beef grading applications. These findings demonstrate that the proposed framework can maintain high grading accuracy under realistic dynamic imaging conditions, supporting its potential for real-time industrial beef quality inspection and automated grading systems.

### 4.6. Discussion and Limitations

The experimental results demonstrate that the proposed framework provides robust and reliable performance for beef quality assessment by jointly analyzing fat marbling distribution and lean-meat color characteristics. The combination of homomorphic filtering and curvelet-based segmentation effectively suppresses frost-induced interference while preserving the directional and structural information of marbling patterns. Furthermore, the integration of chi-square goodness-of-fit analysis and SVM-based color classification enables objective evaluation of marbling uniformity and lean-meat redness, transforming traditionally subjective visual inspection into a quantitative and reproducible grading procedure. The parameter optimization experiments further demonstrate the contribution and sensitivity of each major component, confirming that the adopted parameter configuration provides a favorable balance between detection accuracy, grading performance, and robustness.

The comparison with representative deep-learning models provides additional insight into the strengths of the proposed framework. Under the same evaluation protocol, YOLO-based architectures generally achieved better performance than DenseNet-based models, indicating that spatial localization information contributes to beef quality grading. Nevertheless, the overall grading accuracy of the deep-learning models remained lower than that of the proposed method. Rather than suggesting a universal superiority of feature-based approaches, these results indicate that domain-specific information, such as marbling distribution uniformity and lean-meat color variation, plays an important role in this specialized grading task. The proposed framework explicitly incorporates such knowledge through statistical analysis and interpretable feature extraction, thereby providing both competitive grading performance and enhanced explainability.

From an industrial perspective, the results highlight the importance of combining domain knowledge with machine learning for food-quality inspection. Unlike purely end-to-end approaches, the proposed framework provides transparent decision-making mechanisms that allow the grading process to be traced back to specific quality attributes. Moreover, the dynamic inspection experiments demonstrated that the proposed system maintained grading accuracies above 87.8% at conveyor speeds of 14.80–25.85 cm/s, confirming its feasibility for medium-speed industrial inspection environments. These findings suggest that interpretable hybrid frameworks can provide a practical balance between grading accuracy, robustness, and operational reliability in real-world applications.

Despite these encouraging results, several limitations should be acknowledged. First, although the major parameters were selected through systematic optimization experiments, the current framework still relies on predefined parameter settings that may require adjustment when applied to substantially different imaging conditions, beef cuts, or acquisition systems. Second, while homomorphic filtering effectively reduces frost-related interference, performance may deteriorate under severe contamination, extreme illumination conditions, or excessive surface reflections. Third, the proposed framework relies primarily on two-dimensional RGB imagery and does not exploit depth, volumetric, or spectral information that may further enhance marbling characterization and quality assessment. In addition, motion blur remains a limiting factor in high-speed conveyor-based inspection scenarios.

Future research will focus on improving the adaptability, scalability, and generalization capability of the proposed framework. Potential directions include the development of adaptive parameter optimization techniques, the integration of high-speed imaging hardware to reduce motion blur, and the incorporation of explainable deep-learning components to enhance feature representation while maintaining interpretability. Furthermore, extending the framework to multimodal sensing environments, such as RGB-depth imaging or hyperspectral vision systems, may provide more comprehensive quality information and further improve grading accuracy and robustness in industrial applications.

## 5. Conclusions

This study proposes a vision-based framework for automated beef quality grading by integrating homomorphic filtering, curvelet-based marbling segmentation, chi-square statistical analysis, and SVM-based lean-meat color classification. The proposed framework effectively addresses several challenges encountered in frozen beef inspection, particularly frost-induced interference that complicates accurate fat segmentation and quality evaluation. Through the combination of frequency-domain preprocessing and directional feature extraction, the method achieved robust marbling segmentation while preserving important structural characteristics of fat distribution. Systematic parameter optimization experiments further confirmed the contribution and sensitivity of the major components within the proposed framework, resulting in a reliable and reproducible grading methodology.

Beyond fat segmentation, the proposed framework quantitatively evaluates marbling distribution uniformity and lean-meat color characteristics, enabling a comprehensive and interpretable assessment of beef quality. Comparative experiments showed that domain-specific features, including marbling uniformity and color variation, provide valuable information for quality grading and complement purely data-driven approaches. Furthermore, the dynamic inspection experiments demonstrated that the proposed framework maintained stable grading performance under conveyor-based operating conditions, supporting its feasibility for medium-speed industrial inspection applications.

Overall, the proposed framework provides a reliable, interpretable, and practically applicable solution for automated beef quality grading. The results suggest that integrating frequency-domain image processing, statistical analysis, and machine-learning techniques can effectively support objective food-quality evaluation. Future work will focus on adaptive parameter optimization, explainable deep-learning integration, and multimodal sensing technologies, such as RGB-depth and hyperspectral imaging, to further enhance grading accuracy, robustness, and scalability in industrial environments.

## Figures and Tables

**Table 1:** Class distribution of the dataset used in the large-sample experiment, showing balanced representation across quality grades A, B, and C in all data splits.

Dataset Split	Grade A	Grade B	Grade C	Total Images
Training set	210	210	210	630
Validation set	70	70	70	210
Testing set	70	70	70	210
Total	350	350	350	1050

**Table 2:** Analysis of fat uniformity and lean redness for the front and back sides of the test steak.

**Front Side of Steak**			
**Fat Uniformity**		**Lean Redness**	
Number of uniform fat regions	5	Bright red grids	120
		Moderate red grids	26
Number of fat accumulation regions	3	Dark red grids	9
		Total grids	155
Fat classification	Moderately uniform	Lean classification	Bright red
Fat uniformity (%)	62.5% (5/8)	Lean redness (%)	77.4% (120/155)
Beef Quality Score:	0.6 × 0.625 + (1 − 0.6) × 0.774 = 0.6846 (Grade 6)		
**Back Side of Steak**			
**Fat Uniformity**		**Lean Redness**	
Number of uniform fat regions	4	Bright red grids	132
		Moderate red grids	18
Number of fat accumulation regions	4	Dark red grids	7
		Total grids	157
Fat classification	Moderately uniform	Lean classification	Bright red
Fat uniformity (%)	50.0% (4/8)	Lean redness (%)	84.1% (132/157)
Beef Quality Score:	0.6 × 0.500 + (1 − 0.6) × 0.841 = 0.6364 (Grade 6)		

Note: The final steak grade is determined by the lower of the front and back scores, ensuring a conservative and robust quality assessment.

**Table 3:** Performance evaluation of fat detection under different high-frequency ranges of homomorphic filtering.

High-Frequency Range	*h* = 1.3	*h* = 1.4	*h* = 1.5	*h* = 1.6	*h* = 1.7
(1 − β) %	88.14	88.80	92.68	89.52	89.60
α %	3.54	3.77	4.96	4.03	4.08
CR %	94.65	94.55	94.09	94.43	94.39

**Table 4:** Performance evaluation results for different curvelet transform decomposition levels.

Curvelet Transform Decomposition Level	3 Levels	4 Levels	5 Levels	6 Levels
Detection rate, (1 − β) (%)	91.06	92.06	92.68	91.06
False positive rate, α (%)	4.43	5.43	4.97	5.43
Correct classification rate, CR (%)	94.42	94.42	94.09	94.42

**Table 5:** Performance evaluation results under different binarization threshold values *N*.

Threshold Range	*N* = 1.65	*N* = 1.7	*N* = 1.75	*N* = 1.8	*N* = 1.85
(1 − β)%	94.63	93.72	92.68	92.90	90.29
α%	6.31	5.60	4.97	5.26	3.91
CR%	92.23	93.72	94.09	93.89	94.63

**Table 6:** Detection results under different numbers of partitioned regions for steak fat uniformity analysis.

	Regions	4 Regions	6 Regions	8 Regions	10 Regions	12 Regions	Manual Inspection
Results							
Number of uniform regions		0	3	6	4	5	Densely uniform
Number of non-uniform regions		4	3	2	6	7	
Fat distribution uniformity grade		Fat accumulation	Moderately uniform	Densely uniform	Fat accumulation	Moderately uniform	

**Table 7:** Parameter settings of the SVM model used in this study.

Component	Parameter Setting
Input layer	6 features (lean meat color features: *μ_R_*, *μ_G_*, *μ_B_*, *σ_R_*, *σ_G_*, *σ_B_*)
Cost C	Default: 8 (additional values: 2, 4, 16, 32; total 5 settings)
Gamma *γ*	Default: 2 (additional values: 0.5, 1, 4, 8; total 5 settings)
Output layer	3 classes (bright red *Y*_1_, moderate red *Y*_2_, dark red *Y*_3_)

**Table 8:** Experimental results of classification accuracy for the SVM model with various combinations of hyperparameters C and γ.

	*C*	2	4	8	16	32
*γ*						
0.5		72.5%	72.5%	77.5%	80.0%	80.0%
1		75.0%	77.5%	75.0%	80.0%	47.5%
2		75.0%	75.0%	87.5%	75.0%	70.0%
4		72.5%	72.5%	72.5%	75.0%	75.0%
8		72.5%	72.5%	72.5%	72.5%	72.5%

**Table 9:** Confusion matrix between SVM classification results and human evaluation.

	SVM Classifier	Bright Red	Moderate Red	Dark Red	Total
Human Judgment					
Bright Red		70	2	0	72
Moderate Red		4	74	0	78
Dark Red		0	1	59	60
Total		74	77	59	210
Overall Accuracy		96.67%			

**Table 10:** Precision, Recall, and F1-Score of SVM Classification.

Class	Precision (%)	Recall (%)	F1-Score (%)
Bright Red	94.59	97.22	95.89
Moderate Red	96.10	94.87	95.48
Dark Red	100.00	98.33	99.16
Macro Average	96.90	96.81	96.84
Weighted Avg.	96.73	96.67	96.68

**Table 11:** Quantitative performance comparison of different fat detection methods.

	Method	Spatial Domain		Frequency Domain		
Index		Otsu Method	Watershed Method	Wavelet + Interval Filtering	Curvelet Segmentation	Homomorphic Filtering + Curvelet Segmentation
Fat detection rate, (1 − β) (%)		99.77	71.49	95.24	84.11	92.68
Lean meat misclassification rate, α (%)		41.24	36.31	63.77	2.54	4.97
Correct classification rate, CR (%)		62.97	64.55	43.14	95.12	94.09
Processing time (s)		0.35	0.42	0.69	0.91	1.48

**Table 12:** Comparative performance of deep learning models and the proposed grading framework for direct beef quality grading under identical five-fold cross-validation settings. Values are reported as mean ± standard deviation (%) across the five folds.

	Method	DenseNet 121	DenseNet 201	YOLOv8	YOLOv11	YOLOv26	Proposed Method
Index							
Accuracy (%) (Mean ± SD)		62.65 (±14.25)	63.99 (±13.88)	74.03 (±1.65)	72.22 (±4.39)	75.43 (±1.56)	90.38 (±3.86)
Precision (%) (Mean ± SD)		48.38 (±12.54)	50.15 (±11.96)	66.37 (±9.24)	59.50 (±6.61)	62.20 (±5.96)	89.98 (±4.57)
Recall (%) (Mean ± SD)		43.16 (±8.07)	49.16 (±7.86)	50.25 (±7.51)	54.40 (±6.47)	54.42 (±7.32)	87.68 (±5.14)
F1-Score (%) (Mean ± SD)		42.25 (±8.62)	45.25 (±7.33)	55.27 (±6.61)	54.07 (±3.10)	56.35 (±6.30)	88.81 (±4.85)

Note: All methods were evaluated using the same five-fold cross-validation partitions. Accuracy, Precision, Recall, and F1-score are reported as the average performance across the five folds. The proposed method combines image enhancement, fat marbling distribution analysis, and lean-meat color classification, whereas the deep learning models perform direct image-based quality grading.

**Table 13:** Performance evaluation results of beef steak grading under different conveyor speeds using static grading parameters.

Conveyor Speed Level	Speed 40	Speed 50	Speed 60	Speed 70	Speed 80
Actual speed (cm/s)	14.80	20.75	25.85	28.63	29.98
Image acquisition interval (s)	2.0	1.5	1.0	0.5	0.3
Accuracy (%)	89.45	88.38	87.82	85.53	83.75
Precision (%)	88.98	87.98	87.09	84.22	82.98
Recall (%)	86.68	85.68	85.45	83.75	81.68
F1-Score (%)	87.81	86.81	86.25	83.93	82.31

## Data Availability

The data will be made available on request.
